# A Functional MiR-124 Binding-Site Polymorphism in IQGAP1 Affects Human Cognitive Performance

**DOI:** 10.1371/journal.pone.0107065

**Published:** 2014-09-15

**Authors:** Lixin Yang, Rui Zhang, Ming Li, Xujun Wu, Jianhong Wang, Lin Huang, Xiaodong Shi, Qingwei Li, Bing Su

**Affiliations:** 1 State Key Laboratory of Genetic Resources and Evolution, Kunming Institute of Zoology, Chinese Academy of Sciences, Kunming, China; 2 Laboratory of Primate Neuroscience Kunming Institute of Zoology, Chinese Academy of Sciences, Kunming, China; 3 Department of Biochemistry, Qujing Normal University, Qujing, China; 4 College of Life Science, Liaoning Normal University, Dalian, China; 5 Kunming College of Life Science, University of Chinese Academy of Sciences, Beijing, China; Vanderbilt University Medical Center, United States of America

## Abstract

As a product of the unique evolution of the human brain, human cognitive performance is largely a collection of heritable traits. Rather surprisingly, to date there have been no reported cases to highlight genes that underwent adaptive evolution in humans and which carry polymorphisms that have a marked effect on cognitive performance. IQ motif containing GTPase activating protein 1 (IQGAP1), a scaffold protein, affects learning and memory in a dose-dependent manner. Its expression is regulated by miR-124 through the binding sites in the 3′UTR, where a SNP (rs1042538) exists in the core-binding motif. Here we showed that this SNP can influence the miR-target interaction both *in vitro* and *in vivo*. Individuals carrying the derived T alleles have higher IQGAP1 expression in the brain as compared to the ancestral A allele carriers. We observed a significant and male-specific association between rs1042538 and tactile performances in two independent cohorts. Males with the derived allele displayed higher tactual performances as compared to those with the ancestral allele. Furthermore, we found a highly diverged allele-frequency distribution of rs1042538 among world human populations, likely caused by natural selection and/or recent population expansion. These results suggest that current human populations still carry sequence variations that affect cognitive performances and that these genetic variants may likely have been subject to comparatively recent natural selection.

## Introduction

MicroRNAs (miRNAs) are endogenous ∼21 nt RNAs that play important roles in a variety of biological processes [1]. Animal miRNAs function as guide molecules by imperfect base pairing with hundreds of mRNAs, leading to mRNA translational repression, cleavage or destabilization [2]. The position 2–7 of the mature miRNAs is critical for target recognition, which is defined as the “seed region” of a mature miRNA [3]. Likewise, the pairing status to the remainder of the mature miRNA may also affect binding specificity and affinity [4], [5]. A previous genome-wide scan of miRNA binding site polymorphisms revealed a number of SNPs that may have been subject to recent positive selection throughout several human populations. Among these, rs1042538 (an A to T substitution) showed suggestive evidence of recent positive selection in an African population (Yoruba in Ibadan, YRI) [6]. This SNP at the position 1 of the miR-124 binding site in the 3′UTR of IQGAP1 gene and it is highly conserved in major non-human primate lineages, including chimpanzee, gibbon, rhesus macaque and common marmoset (www.rhesusbase.org; http://asia.ensembl.org). Hence, it may affect the binding affinity of miR-124, and eventually lead to possible functional consequences.

MiR-124 is one of the most conserved and abundantly expressed neuron specific miRNAs. It plays a critical role in neuronal differentiation and proper nervous system development [7]–[9]. As a potential target gene of miR-124, IQGAP1 is a widely expressed scaffold protein and is engaged in multiple fundamental cellular activities, such as cell adhesion, cell migration and regulation of cytoskeleton [10]. More importantly, IQGAP1 is known to have neuron specific functions and is involved in learning and memory [11]. For example, IQGAP1 affects memory formation in a dose-dependent manner through the N-cadherin/cytoskeletal IQGAP1/Erk signaling pathway [12]. Likewise, IQGAP1 knockout mice exhibit marked long-term memory deficits accompanied by an impaired hippocampal long-term potentiation (LTP) [13]. An earlier *in vitro* experiment showed that the reporter gene bearing the 3′UTR of IQGAP1 could be down-regulated by miR-124 [14]. Additionally, IQGAP1 and miR-124 are co-expressed in neuronal cells [13], [15], suggesting that IQGAP1 may be a direct target of miR-124 in the brain.

Based on these previous observations, we hypothesized that the presence of the SNP rs1042538 might alter miR-124's regulation of IQGAP1, and in doing so, exert some effect on cognitive performance across human populations. To test this supposition, we first tested whether this SNP affects miR-124 IQGAP1 interaction *in vitro* and *in vivo*. Results of our analysis showed that this was indeed the case, so we opted to further see if there was a connection between this SNP and cognitive performance. An analysis of two independent Chinese cohorts showed a significant association between this SNP and cognitive performance in males, indicating that rs1042538 does have some functional role to play in cognitive ability. Finally, we examined the evolutionary history of rs1042538, we found a high level population differentiation of allele frequency, and we demonstrated that the genomic region containing rs1042538 is likely subject to recent positive selection.

## Material and Methods

### Plasmid construction and reporter gene assay

We cloned IQGAP1 3′UTR fragment of 975 bp covering three putative miR-124 binding sites and inserted it into the downstream of firefly luciferase reporter gene. In brief, we amplified the 3′UTR fragment from the human genomic DNA by PCR. The PCR product, which contained the primer-introduced Bgl Π and Xba Ι sites, was digested, purified, and cloned into pGL 3 firefly luciferase reporter plasmid. Finally, we created three reporter gene constructs: (1) IQGAP1 3′UTR with the ancestral allele (rs1042538/A), (2) IQGAP1 3′UTR with the derived allele (rs1042538/T), (3) IQGAP1 3′UTR with the site-directed A to T mutation at the rs1042538 site. The sequences of the constructs were confirmed by Sanger sequencing.

The miR-124 and control miRNA duplexes were synthesized in Shanghai GenePharma. The duplexes sequences were as follows:

miR-124 (as previously described [14])

UAAGGCACGCGGUGAAUGCCA/GCAUUCACCGCGUGCCUUAAU

control miRNA

UUCUCCGAACGU GUCACGUTT/ACGUGACACGUUCGGAGAATT

HEK293T cells were grown in DMEM containing 10% FBS (Thermo Fisher, USA). When cells were grown to a confluence of 40–60% in 24-well plates, they were transfected with 100 ng pGL3 firefly luciferase report plasmid, 200 ng pRL-TK renilla luciferase plasmid and 20 pmol miRNA using Lipofectamine 2000 (Invitrogen, USA). All experiments were performed in quadruplicate. The activities of the two luciferases were measured 36 h after transfection using the Dual-Luciferase Reporter Assay System (Promega, USA). The firefly activity has been normalized to renilla.

### Quantitative measurement of miR-124, IQGAP1 mRNA and protein expression

DNA was extracted from 60 frozen human parietal cortex tissues with proteinase-K-chloroform method. Next, rs1042538 was genotyped using the SNaPshot method (Applied Biosysterm, USA). Total RNA was extracted using TRIzol (Invitrogen, USA). Reverse transcription was performed using the miScript PCR Starter Kit (Qiagen, Germany). Real-time quantitative PCR was performed using gene specific primers, and the fold change of gene expression levels was calculated using the Double standard curve method. The GAPDH was used as the internal control. Mature miRNA expression was quantified using SYBR Green master mix (TaKaRa, Japan) and miR Reverse Primer Kit (Guangzhou RiboBio, China). Small nuclear RNA U6 snRNA was used as internal control. The relative quantification was calculated using the 2^−ΔCt^ method.

Total proteins were extracted using RIPA lysis buffer, and quantified using a Pierce bicinchoninic acid protein assay. Proteins were analyzed by western blot with antibodies against IQGAP1 (Abcam, ab56529, 1∶100) or actin (Abcam, ab3280, 1∶5000). Immunoreactivity was detected with a chemiluminescence system. The band intensity was analyzed using ImageJ analyzer software.

### Sample collection and statistical analyses for association studies

We recruited 195 (89 males) healthy undergraduate students from Qujing Normal University to participate in three sub-tests of Chinese Wechsler Memory Scale-revised (WMS-CR). The mean age of the participants was 20.88±1.53 y (range from 18 to 25). Most participants (96%) are Han Chinese and few participants (4%) are Chinese ethnic minority (CEM), all self-reported as having no neurological or psychiatric history. For replication test, we recruited an independent cohort from Liaoning Normal University, including 265 (140 males) individuals, with a mean age of 19±0.96 y (range from 16 to 23). All DNA samples were extracted from blood. Genotyping of rs1042538 was conducted using the SNaPshot method And the Hardy-Weinberg equilibrium of this SNP was assessed using Haploview (version 4.2) [16]. The association analysis was conducted using SPSS (version 17.0) statistical software. Meta-analyses of the two cohorts were conducted by RevMan 4.3.

### Post mortem brain tissue collection

Human brain tissues were provided by Chinese Brain Bank Center (CBBC, http://cbbc.scuec.edu.cn, Wuhan, China). According to the protocol of CBBC, written informed consent for brain autopsy and use of the brain tissue for research was obtained from either the donors or their relatives. These donors did not have neurological disease. The sample information is given in Table S1.

### Ethical approval

The protocol of this study was approved by the internal review board of Kunming Institute of Zoology, Chinese Academy of Sciences (approval ID, SWYX - 2010–002).

### WMS-CR test

We used WMS-CR to measure memory functions [17]. The same person preformed all tests in order to avoid variations by different experimenters. The test was administered in accordance with the instructions in the manual. All subjects sat before a desk, 150 cm away from the experimenter in a sound-attenuated room. The picture recall test and verbal association test consisted of a learning phase and a recognition phase. In the learning phase of picture recall test, participants were instructed to learn and memorized 20 target pictures printed in a card that lasted approximately 90 s. In the recognition phase, experimenter took away that card and let the participants recall the pictures. The learning phase of verbal association test contained 10 pairs of Chinese words printed in ten cards respectively. These cards were presented for 2 s to participants in sequence. Then the administrator informed participants of the first word on the card and allowed the subjects to recall the second word in the same card after 5 s. The Tactual Performance Test (TPT) is a tactile-kinesthetic problem solving and learning and memory task [18] which was revised in Chinese Wechsler memory Scale in 1989 by Gong Y X [19]. The variety of abilities involved in this task makes it a useful measure of brain development and cognitive status. The TPT makes use of a formboard with 9 cut-out space and wooden blocks to fit into the spaces. The standard administration procedure is to blindfold the subject prior to his/her seeing the board. Then the subject places the blocks onto the formboard three times, first with the dominant hand, next with the non-dominant hand, and then with both hands. Administrators record the time required for each operation, and add the three time values together to obtain a total time for placing the blocks on the board. The formboard is then removed from view of the subject, the subject's blindfold is removed, and without prior warning, he/her is asked to draw a diagram of board, including as many shapes as possible and in correct location relative to each other. Usually three scores are obtained from this procedure: (1) total time to place the blocks on the board (time); (2) number of shapes correctly recalled (memory); (3) number of correctly recalled shapes that also are correctly located in the subject's reproduction of the formboard (location). Although three different scores are obtained, they are not independent. This is especially true for Memory and Location, because the number correctly located is dependent upon the number recalled. The three scores are entered into a formula calculate a value [20].

### Global distributions of rs1042538 and population genetics analyses

Global distributions of rs1042538 in major world populations were extracted from the HGDP Selection Browser (http://hgdp.uchicago.edu). For 1000 Human Genome data analyses, we obtained the resequencing data of 460 individuals for the surrounding regions of rs1042538 (chr15:91044408) from the 1000-Human-Genome Project website (http://www.1000genomes.org). Individuals from three population groups were analyzed: Europeans (CEU); East Asians (ESA)—which includes Han Chinese in Beijing (CHB), Southern Han Chinese (CHS) and Japanese in Tokyo (JPT)—and Yoruba in Ibadan (YRI). Neutrality test was conducted with DnaSP 5.10 [21] using the entire IQGAP1 3′UTR (chr.15:91043408–91045408) with the Africans (YRI) used as an out-group [22]–[25]. The median-joining network was constructed with NETWORK 4.5 [26] using SNPs within 10 kb up- and down- stream of rs1042538. EHH values were calculated with Sweep software [27] using SNPs within 500 kb up- and down- stream of rs1042538.

## Results

### Rs1042538 affects miR-124 IQGAP1 interaction

**Figure 1:**
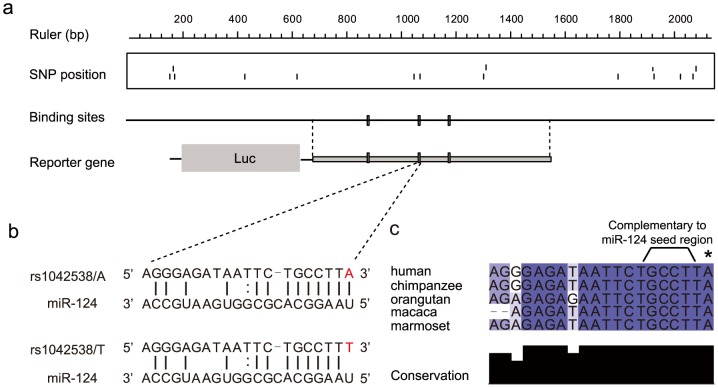
MiR-124-IQGAP1 3′UTR interaction. (**a**) Three putative miR-124 binding sites were found in the 3′UTR of IQGAP1 using TargetScanS algorithm. All SNPs located at the 3′UTR and the fragment cloned for reporter assay was also shown. (**b**) rs1042538 (highlighted in red) is located at position 1 of the second miR-124 binding site and the derived T allele disrupts an A:U Waston Crick pairing. (**c**) Conservation of the second miR-124 binding site among diverse primate species. The position of rs1042538 is marked by an asterisk.

**Figure 2:**
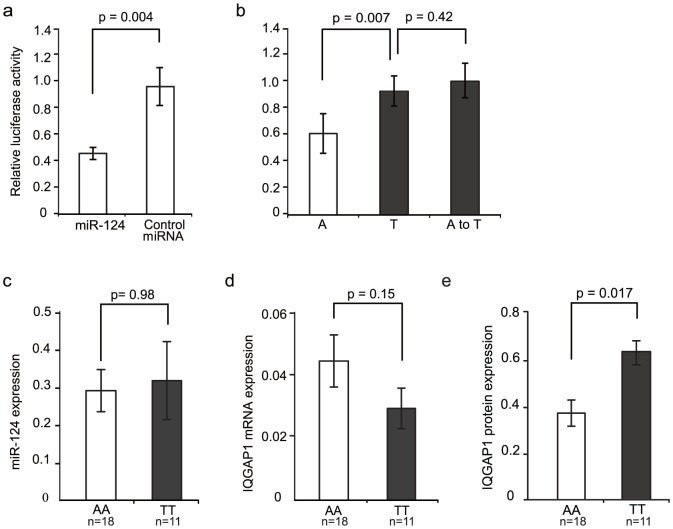
Modulation of miR-124 regulation of IQGAP1 by rs1042538 *in vitro* and *in vivo*. (**a**) Luciferase activity of IQGAP1 reporter gene harboring the ancestral A allele (refers to Luc-A) was significantly reduced when co-transfected with miR-124 as compared to the control miRNA. Error bars denote s.d. for quadruplicate experiments. (**b**) Luc-A has a lower activity than the reporter gene harboring the derived T allele (refers to Luc-T) when co-transfected with miR-124. When the rs1042538 position “A” of Luc-A was mutated to a “T” (refers to Luc-A-to-T), the activity of Luc-A-to-T is similar to Luc-T. Error bars denote s.d. for quadruplicate experiments. (**c**) Mature miR-124 expression level in human brain parietal cortex tissue is similar between the AA and the TT genotypes. U6 snRNA was used as internal control. (**d**) The mRNA expression of IQGAP1 in human brain parietal cortex tissue is similar between the AA and TT genotypes. GAPDH was used as internal control. (**e**) Protein expression quantification based on the result of western blot. Actin was used as loading control.

There are three putative miR-124 binding sites in the 3′UTR of IQGAP1 (Figure. 1a). The derived T allele of rs1042538 is located at position 1 of the second binding site and expected to disrupt an A:U Watson-Crick pairing of the ancestral A allele (Figure. 1b). The binding sites complementary to the miR-124 seed region (position 2–7), as well as position 1, are totally conserved across all major nonhuman primate lineages (New World monkey, Old World monkey and great apes) (Figure. 1c), implying its functional constraint over the course of primate evolution. To validate if IQGAP1 is indeed a miR-124 target and if the two alleles have differentiated miRNA regulations, we constructed three reporter plasmid by fusing the IQGAP1 3′UTR into the downstream of the luc reporter gene (Figure.1a). The first plasmid contained the ancestral A allele of rs1042538, the second contained the derived T allele of rs1042538, while the third is the same as the first plasmid except for an A-to-T site-specific mutation of the ancestral A allele of rs1042538. We transfected the first plasmid into HEK293T cells either with a miR-124 mimic or control miRNA, and the luciferase activity was significantly reduced as compared to the miR-124 transfection with the control miRNA transfection (p = 0.004, Student's t-test) (Figure. 2a), indicating that IQGAP1 can be targeted by miR-124. We next transfected all three plasmids with miR-124 mimic, and as expected, the T allele caused a significant impairment of the miR-target interaction as compared to the A allele (p = 0.007, Student's t-test) (Figure. 2b). The luciferase activity of the A-to-T mutation plasmid was also the same as the T allelic plasmid, ruling out the possibility that the observed difference may have been caused by other hidden mutations in the constructed plasmid (Figure. 2b). Collectively, these findings demonstrate that IQGAP1 is a true target of miR-124, and that the T allele at rs1042538 can impair the interaction between miR-124 and IQGAP1.

We next examined if the T allele could impair miR-124 IQGAP1 interaction *in vivo*. Using tissue samples from 29 human parietal cortices (18 AA homozygote individuals and 11 TT homozygote individuals), we measured the mature miR-124 expression and IQGAP1 expression at both mRNA and protein levels. We found that miR-124 was equally expressed in both genotypes (Figure. 2c) (Student's t-test, p = 0.98), and no mRNA expression difference was detected for IQGAP1 either (Figure. 2d) (Student's t-test, p = 0.15). However, a significantly higher expression of the TT genotype was detected as compared with the AA genotype (Student's t-test, p = 0.017) (Western Blot result in Figure S1., statistical test in Figure. 2e). Taken together, the results indicate that the derived T allele of rs1042538 can impair miR-124 IQGAP1 interaction in vivo and leads to an increased expression of IQGAP1 proteins in the brain. A previous report observed a similar effect of this SNP on IQGAP1 in breast samples [28].

### Association of IQGAP1 rs1042538 with cognitive performance

**Figure 3:**
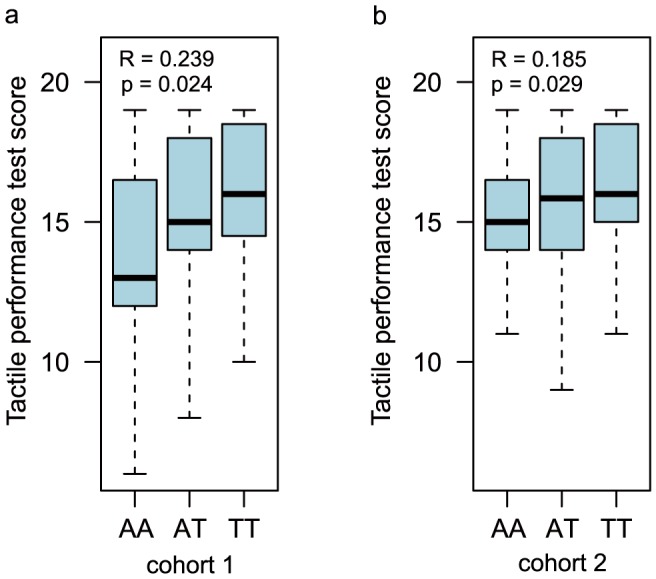
The association between tactual performance test score and rs1042538 genotype. Box plot of tactual performance test scores in Qujing male cohort (a) and Liaoning male cohort (b). Pearson's R and p value were indicated.

The known involvement of both miR-124 and IQGAP1 in cognitive performance led us to hypothesize that this SNP may have functional consequence to human cognition. We accordingly conducted an association analysis. We first recruited 195 healthy volunteers (college students, 89 males and 106 females, 20.88±1.53 years old) from Qujing Normal University in southwestern China. We genotyped these individuals for the rs1042538 SNP, and we identified 60 AA homozygotes, 91 AT heterozygotes and 44 TT homozygotes (Table 1). The genotype distribution is within the Hardy-Weinberg equilibrium (p = 0.377, Chi-square test). All subjects underwent three sub-tests of WMS-RC, including a picture recall test, a verbal association test and a tactual performance test (score distributions of the three sub-tests are shown in Table S2, and Figure S2). We then conducted association analysis under an additive model using linear regression. When both the males and female samples were pooled together, no association was observed between cognitive performance and rs1042538 (data not shown). This is not entirely unexpected, as males and females are known to perform differently for learning and memory tests. For example, males score better on tests of spatial abilities [29]–[31] and tactual performance tests [32]. To remove the effect of gender stratification, males and females were analyzed separately. This analysis showed a significant association in males for tactual performance (p = 0.024, R = 0.239) (Figure 3a), but not for either picture recall or verbal association. No association was observed for all sub-tests among the female subjects (Table 1).

**Table 1:** Demographic characteristics and working memory performance of Chinese subjects from Qujing Normal University.

	Males			Females				
	A/A	A/T	T/T	P-value	A/A	A/T	T/T	P-value
Number of subjects	28	42	19		32	49	25	
Age(SD)	20.67(1.53)	21.14(1.34)	21.05(1.40)		20.9(1.40)	20.72(1.39)	21(1.43)	
Education years(SD)	14.57(0.55)	14.61(0.50)	14.3(0.43)		14.21(1.00)	14.04(1.01)	14.46(1.07)	
WMS-CR								
Picture recall score (SD)	11.76(1.33)	12.38(1.37)	12.11(1.65)	0.583	12.68(1.50)	12.5(1.40)	12.33(0.87)	0.268
Verbal association score (SD)	13.06(2.56)	13.96(2.68)	13.39(2.40)	0.418	13.7(2.74)	13.72(2.38)	12.99(2.04)	0.345
Tactual performance score (SD)	13.29(3.56)	15.1(3.23)	15.89(3.15)	**0.024**	14.19(3.34)	14.33(2.91)	13.54(3.97)	0.786

Note: The bold number indicates a statistically significant P-value.

As such a small sample can often lead to confounding results or false positives, to replicate and verify the initial findings, we recruited an independent cohort of 265 college students (140 males and 125 females, 19±0.96 years old) from Liaoning Normal University of northeastern China. In this sample, we identified 80 AA homozygotes, 127 A/T heterozygotes and 58 TT homozygotes (Table 2), leaving the genotype distribution also in line with the Hardy-Weinberg equilibrium (p = 0.57, Chi-square test). Using the same method as before, the 265 subjects were tested for tactual performance only (score distribution of the test is shown in Table S2 and Figure S2). This time, we also found a significant association between tactual performance score and genotype in males (p = 0.029, R = 0.185) but not in females (Table 2, Figure 3b). Meta-analysis of the combined independent samples from both Chinese universities indicated an even more significant association (p = 0.002, R = 0.206) in males, with no observed heterogeneity between these two cohorts (p>0.1, Q test). On the whole, each separate analyses as well as the joint meta-analysis showed that the derived T allele confers higher tactual performance in males.

**Table 2:** Replication of tactual performance of Chinese subjects from Liaoning Normal University.

	Males			Females				
	A/A	A/T	T/T	P-value	A/A	A/T	T/T	P-value
Number of subjects	44	65	31		36	62	27	
Age(SD)	18.62(1.22)	18.67(0.86)	18.18(0.68)		17.85(0.72)	18.4 (0.88)	18.8(1.04)	
Tactual performance score (SD)	15.05(2.15)	15.6(2.51)	16.32(2.57)	**0.029**	15.36(3.37)	15.02(2.45)	16.41(2.68)	0.186

Note: The bold number indicates a statistically significant P value.

### Worldwide allele-frequency distribution and evolutionary analyses of rs1042538

**Figure 4:**
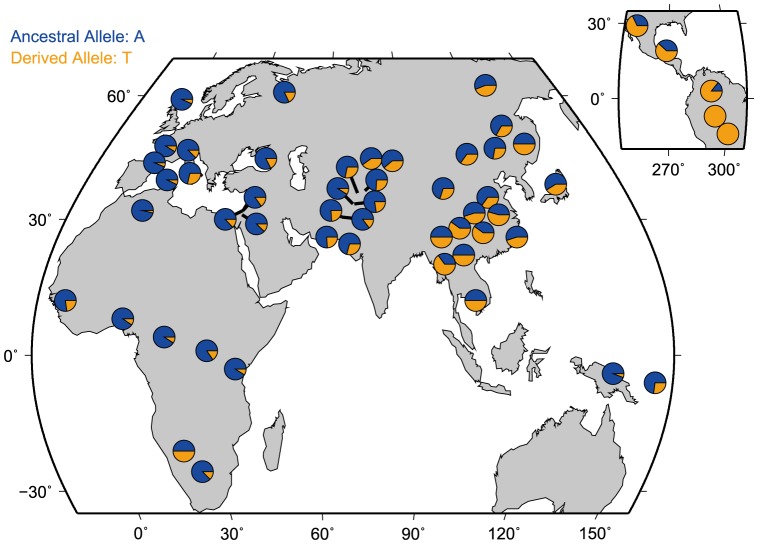
Worldwide distribution of the allele frequency of the SNP rs1042538.

**Figure 5:**
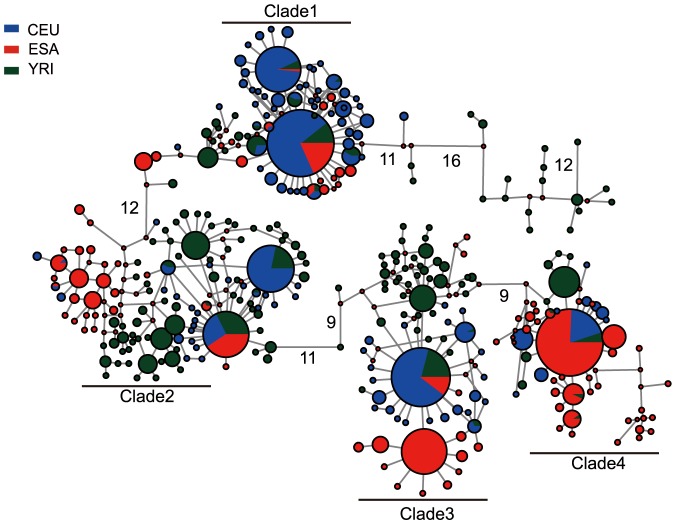
Median-joining network of haplotype genealogy in three major world populations. Network was constructed using sequencing data obtained from 1000 Human Genomes project. We used 10 Kb upstream and downstream of rs1042538 (chr15: 91044408) for analysis. The size of circle is proportional to the frequency of the haplotype.

**Figure 6:**
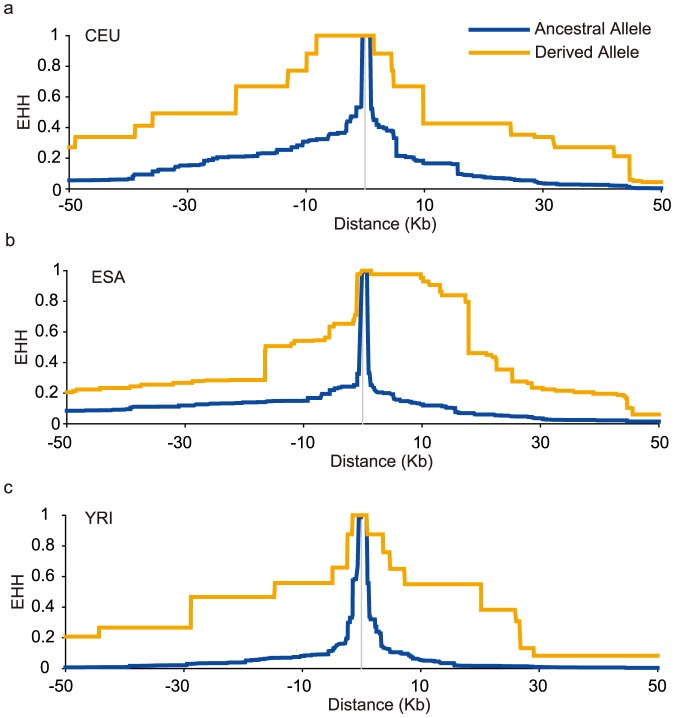
Recent expansion for the derived allele of rs1042538 in three populations. The expanded haplotype homozygosity (EHH) for both the ancestral and the derived allele in three populations.(**a**) CEU; (b) ESA; (**c**) YRI.

In order to further understand the evolutionary history of rs1042538, we first examined the allele frequency distribution of rs1042538 among global populations. We found large allelic differences among 53 different populations (Figure. 4). Generally, the derived allele (T) of rs1042538 is dominant (>50%) in East Asian (ESA) populations, reaching fixation in some of the surveyed American Indian populations. Moving west, the derived allele becomes relatively less prevalent in Central Asia as well as in southern Africa, and then only rarely occurs in northern Africa, the Middle East and Europe (Figure. 4). The observed population differentiation at this locus can largely be attributed to neutral drift, population structure, or Darwinian positive selection [33]. To determine the driving force, then we used data from the 1000 Human Genomes project that contained whole genome sequencing data from five distinct ethnic populations (CEU, CHB, CHS, JPT and YRI). For this particular study, we applied several different methods for detecting selection. While the traditional allele frequency based tests are useful in detecting relatively old selection they have a comparatively low power to detect recent selection [34] and the haplotype based methods are more powerful to detect recent selection [34], [35]. We first performed traditional neutrality tests (i.e., Tajima's D, Fu and Li's D, F, and Fay and Wu's H). We also used the entire 3′UTR region of IQGAP1 (2 kb, chr15: 91043408–91045408, Hg19 version) to perform coalescent simulations that incorporate demographic scenarios. None of these tests showed any significant deviation from the expected neutral expectation (Table S3). We subsequently performed the haplotype based tests by first constructing a median-joining network of haplotype genealogy (Figure.5). The topology of the median-joining network indicates there are four major clades, all of which have star-like shapes, which suggests a recent population expansion. In Clade3, there was an ESA specific haplotype accounts for 34.3% of all the haplotypes in ESA, but which was absent in non-ESA populations (Figure. 5), suggesting a recent positive selection on this genomic region covering rs1042538. We also performed the long-range haplotype test [35] and found highly extended haplotype homozygosity (EHH) for the derived allele of rs1042538 in major world populations including East Asians (Figure.6), consistent with the previous observation of positive selection [6]. Taken collectively, these results indicate that rs1042538 may have been subject to recent positive selection, leading to the observed allelic difference among the surveyed global populations.

## Discussion

Previously, several SNPs such as rs363039 and rs17070145, which are located at introns, were shown to have some association with human cognition [36], [37] but neither were functional SNPs located at the 3′UTR region of a microRNA target gene. For this study, we opted to focus on rs1042538, a functional variant residing in a miR-124 binding site of the IQGAP1 3′UTR. Previous studies suggest that both IQGAP1 and brain expressed miRNAs are involved in human learning and memory [13], [38], [39]. It has been shown that down-regulation of IQGAP1 can lead to long-term memory deficits [13] and impaired contextual fear memory formation in mice [12]. Meanwhile, the down-regulation of the entire miRNA repertoire in neuron, including miR-124, can enhance learning and memory in mice [38]. It then stands to reason that an increase of IQGAP1 in the brain may enhance certain aspects of cognitive performance. In this study, we demonstrated that individuals carrying the derived T allele of rs1042538, which destroys the miR-124-target interaction and leads to an increased IQGAP1 protein expression, is associated with better tactual performance.

There is one puzzling factor that deserves mention. The observed gender dependent association of tactual performance is rather unusual, because our findings would seem to imply that the manifestation of variations for cognitive performances such as spatial abilities may differ between males and females. One possible explanation may be that numerous studies have confirmed that hormone levels have a strong impact on learning and memory [40] and that females often have comparatively better spatial memory during the nonmenstrual phase [41]. Though our female participants (17–22 years old) were all adults, when we initially used WMS-RC to measure the memory abilities, we did not consider their menstruation status, which may account for the failure to identify association between genotype and tactual performance. There is also the possibility that there is no association between the studied SNP and cognitive performance among females, and that there are other confounding factors in the observed association between tactual performance in males with the derived allele of rs1042538. Clearly, further studies incorporating this factor are needed to give a satisfactory response, but nonetheless, either possibility is quite intriguing from many perspectives. Finally, it should be noted that the recruited college students have presumably higher-than-average intelligence, and whether the observed association still holds for the general population is yet to be tested.

The diverged allele frequency distribution of rs1042538 is intriguing. We observed an ESA specific haplotype, which suggests a recent expansion of this haplotype containing the derived T allele of rs1042538 among East Asian populations. We also observed a higher EHH value for the derived T allele in multiple populations including those from ESA, a signature of recent positive selection on this sequence variation. The observed higher tactual performance by those with the derived T allele provides a possible driving force if Darwinian positive selection has been acting on this SNP in East Asians. Interestingly, another report showed that the derived T allele of this SNP was also associated with a lower risk of developing breast cancer [28]. Aside from cognitive performance, there may then be other traits under selection. It should be noted that as the molecular signature of positive selection is not very strong in East Asians, its prevalence in East Asia may also simply be caused by recent population expansion and genetic drift, though any definitive answer is outside the scope of this study.

In conclusion, our work showed that a SNP residing in the IQGAP1 3′UTR miRNA binding site can alter the affinity of miRNA binding. This SNP is likely a target of positive selection in East Asian populations, and it is associated with cognition performance at least among Chinese populations. Given the abundant SNPs that are capable of either creating or destroying putative miRNA target sites [6], [42], this group of regulatory variants may be important source in the human genome that leads to some interesting phenotypic variations.

## Supporting Information

Figure S1 Comparison of IQGAP1 expression at protein level between AA and TT genotype in human brain parietal cortex tissues.
(DOCX)

Figure S2 The score distributions of three sub-tests.
(DOCX)

Table S1 Information of human brain tissues.
(DOCX)

Table S2 Details of genotype and phenotype data.
(XLSX)

Table S3 Nucleotide diversity and neutrality tests for IQGAP1 (covering rs1042538) in human populations.
(DOCX)
