# Supplementary material for: A Functional MiR-124 Binding-Site Polymorphism in IQGAP1 Affects Human Cognitive Performance
Source: PLoS One. 2014 Sep 15;9(9):e107065. doi: 10.1371/journal.pone.0107065 (PMC4164536; doi:10.1371/journal.pone.0107065)

**Fig S1.** **Comparison of IQGAP1 expression at protein level between AA and TT genotype in human brain parietal cortex tissues.** Actin was used as loading control for western blot analysis.


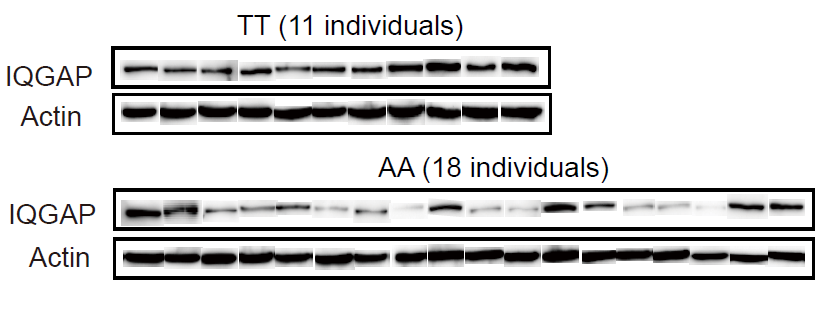

Supplement: Figure S1 — Comparison of IQGAP1 expression at protein level between AA and TT genotype in human brain parietal cortex tissues. (DOCX) [file pone.0107065.s001.docx]
