# Supplementary material for: A Functional MiR-124 Binding-Site Polymorphism in IQGAP1 Affects Human Cognitive Performance
Source: PLoS One. 2014 Sep 15;9(9):e107065. doi: 10.1371/journal.pone.0107065 (PMC4164536; doi:10.1371/journal.pone.0107065)
Supplement: Figure S2 — The score distributions of three sub-tests. (DOCX) [file pone.0107065.s002.docx]

**Fig S2.** **The score distributions of three sub-tests.** (a) TPT score distribution of QuJing population. (b) Verbal association test score distribution of QuJing population. (c) Picture recall test score distribution of QuJing population. (d) TPT score distribution of Liaoning population.
