# Supplementary material for: A Functional MiR-124 Binding-Site Polymorphism in IQGAP1 Affects Human Cognitive Performance
Source: PLoS One. 2014 Sep 15;9(9):e107065. doi: 10.1371/journal.pone.0107065 (PMC4164536; doi:10.1371/journal.pone.0107065)
Supplement: Table S1 — Information of human brain tissues. (DOCX) [file pone.0107065.s003.docx]

**Table S1.** Information of human brain tissues

| Sample ID | Gender | Age | Genotype |
| --- | --- | --- | --- |
| 1 | male | 48Y | AA |
| 2 | female | 1Y | AA |
| 3 | female | 22Y | AA |
| 4 | female | 46Y | AA |
| 5 | male | 62Y | AA |
| 6 | female | 20Y | AA |
| 7 | female | 36Y | AA |
| 8 | male | 47Y | AA |
| 9 | male | 31Y | AA |
| 10 | male | 47D | AA |
| 11 | male | 40Y | AA |
| 12 | male | 5Y | AA |
| 13 | female | 1Y | AA |
| 14 | female | 47Y | AA |
| 15 | male | 47Y | AA |
| 16 | male | 53Y | AA |
| 17 | female | 35Y | AA |
| 18 | male | 22Y | AA |
| 19 | male | 40Y | TT |
| 20 | male | 46Y | TT |
| 21 | male | 55Y | TT |
| 22 | male | 59Y | TT |
| 23 | female | 60Y | TT |
| 24 | female | 49Y | TT |
| 25 | female | 19Y | TT |
| 26 | female | 8M | TT |
| 27 | male | 23Y | TT |
| 28 | female | 48Y | TT |
| 29 | male | 5Y | TT |

Note: The listed 29 human parietal cortex tissues were homozygotes for rs1042538 (either TT or AA), selected by genotyping a total of 60 samples. The remaining 31 samples were heterozygotes for rs1042538, and were not used in protein expression quantification.
