# Supplementary material for: A Functional MiR-124 Binding-Site Polymorphism in IQGAP1 Affects Human Cognitive Performance
Source: PLoS One. 2014 Sep 15;9(9):e107065. doi: 10.1371/journal.pone.0107065 (PMC4164536; doi:10.1371/journal.pone.0107065)
Supplement: Table S3 — Nucleotide diversity and neutrality tests for IQGAP1 (covering rs1042538) in human populations. (DOCX) [file pone.0107065.s005.docx]

**Table S3.** Nucleotide diversity and neutrality tests for IQGAP1 (covering rs1042538) in human populations.

| **Population** | **Sample size** | **Length(bp)** | **Polymorphic sites** | **Π(×10-4)** | **Frequency-spectrum-based tests** | | | | | |
| --- | --- | --- | --- | --- | --- | --- | --- | --- | --- | --- |
|  |  |  |  |  | **Tajima's D** | **Fu and Li's D** | **Fu and Li's F** | **Fay and Wu's H** | |  |
| CEU | 85 | 2000 | 4 | 2.0442 | 1.102 | -0.384 | 0.127 | | 0.532 | |
| EAS | 286 | 2000 | 9 | 8.2152 | 1.106 | 1.1954 | 1.408 | | -0.1624 | |
| YRI | 89 | 2000 | 5 | 2.2463 | 0.265 | -0.148 | -0.01 | | -1.76 | |
